# Estimates for capacity and discrepancy of convex surfaces in sieve-like domains with an application to homogenization

**DOI:** 10.1007/s00526-016-1088-2

**Published:** 2016-11-02

**Authors:** Aram L. Karakhanyan, Martin Strömqvist

**Affiliations:** 1 School of Mathematics, University of Edinburgh, Edinburgh, Scotland, UK; 2 Department of Mathematics, Uppsala University, Uppsala, Sweden

**Keywords:** 35R35, 35B27, 32U15, 11K06

## Abstract

We consider the intersection of a convex surface $\Gamma$ with a periodic perforation of $\mathbb {R}^d$, which looks like a sieve, given by $T_\varepsilon = \bigcup _{k\in \mathbb {Z}^d}\{\varepsilon k+a_\varepsilon T\}$ where *T* is a given compact set and $a_\varepsilon \ll \varepsilon$ is the size of the perforation in the $\varepsilon$-cell $(0, \varepsilon )^d\subset \mathbb {R}^d$. When $\varepsilon$ tends to zero we establish uniform estimates for *p*-capacity, $1<p<d$, of the set $\Gamma \cap T_\varepsilon$. Additionally, we prove that the intersections $\Gamma \cap \{\varepsilon k+a_\varepsilon T\}_k$ are uniformly distributed over $\Gamma$ and give estimates for the discrepancy of the distribution. As an application we show that the thin obstacle problem with the obstacle defined on the intersection of $\Gamma$ and the perforations, in a given bounded domain, is homogenizable when $p<1+\frac{d}{4}$. This result is new even for the classical Laplace operator.

## Introduction

**Fig. 1:**
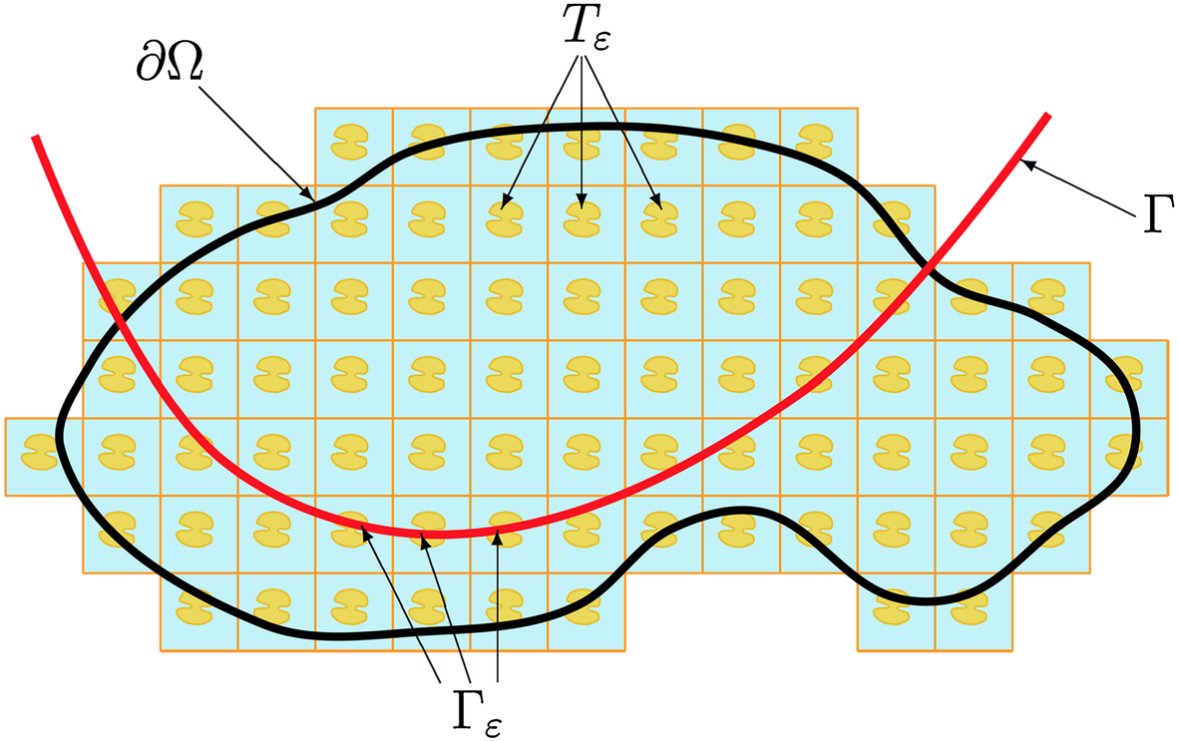
The sieve-like configuration with convex $\Gamma$

In this paper we study the properties of the intersection of a convex surface $\Gamma$ with a periodic perforation of $\mathbb {R}^d$ given by $T_\varepsilon = \bigcup _{k\in \mathbb {Z}^d}\{\varepsilon k+a_\varepsilon T\}$, where *T* is a given compact set and $a_\varepsilon$ is the size of the perforation in the $\varepsilon$-cell. Our primary interest is to obtain good control of *p*-capacity $1<p<d$ and discrepancy of distributions of the components of the intersection $\Gamma \cap T_\varepsilon$ in terms of $\varepsilon$ when the size of perforations tends to zero. As an application of our analysis we get that the thin obstacle problem in periodically perforated domain $\Omega \subset \mathbb {R}^d$ with given strictly convex and $C^2$ smooth surface as the obstacle and *p*-Laplacian as the governing partial differential equation is homgenizable provided that $p<1+\frac{d}{4}$. Moreover, the limit problem admits a variational formulation with one extra term involving the mean capacity, see Theorem 3. The configuration of $\Gamma$, $\Gamma _\varepsilon$, $T_\varepsilon$ and $\Omega$ is illustrated in Fig. 1.

This result is new even for the classical case $p=2$ corresponding to the Laplace operator. Another novelty is contained in the proof of Theorem 2 where we use a version of the method of quasi-uniform continuity developed in [4].

### Statement of the problem

Let

$$\begin{aligned} T_\varepsilon = \bigcup _{k\in \mathbb {Z}^d}\{\varepsilon k+a_\varepsilon T\}, \end{aligned}$$

and let

$$\begin{aligned} \Gamma _\varepsilon = \Gamma \cap T_\varepsilon . \end{aligned}$$

We assume that $\Gamma$ is a strictly convex surface in $\mathbb {R}^d$ that locally admits the representation

1 $$\begin{aligned} \{(x',g(x')):x'\in Q'\}, \end{aligned}$$

where $Q'\subset \mathbb {R}^{d-1}$ is a cube. For example, $\Gamma$ may be a compact convex surface, or may be defined globally as a graph of a convex function.

Without loss of generality we assume that $x_d = g(x')$ because the interchanging of coordinates preserves the structure of the periodic lattice in the definition of $T_\varepsilon$. We will also study homogenization of the thin obstacle problem for the *p*-Laplacian with an obstacle defined on $\Gamma _\varepsilon$. Our goal is to determine the asymptotic behaviour, as $\varepsilon \rightarrow 0$, of the problem

2 $$\begin{aligned} \min \left\{ \int _\Omega |\nabla v|^pdx+\int _\Omega hvdx:v\in W^ {1,p}_0(\Omega )\text { and }v\ge \phi \text { on }\Gamma _\varepsilon \right\} , \end{aligned}$$

for given $h\in L^q(\Omega )$, $1/p+1/q=1$ and $\phi \in W^{1,p}_0(\Omega )\cap L^\infty (\Omega )$.

We make the following assumptions on $\Omega$, *T*, $\Gamma$, *d* and *p*:

- $(A_1)$ $\Omega \subset \mathbb {R}^d$ is a Lipschitz domain.
- $(A_2)$ The compact set *T* from which the holes are constructed must be sufficiently regular in order for the mapping
  $$\begin{aligned} t\mapsto {\text {cap}}(\{\Gamma +t e\}\cap T) \end{aligned}$$
  to be continuous, where *e* is any unit vector. This is satisfied if, for example, *T* has Lipschitz boundary.
- $(A_3)$ The size of the holes is
  $$\begin{aligned} a_\varepsilon =\varepsilon ^{d/(d-p+1)}. \end{aligned}$$
  This is the critical size that gives rise to an interesting effective equation for (2).
- $(A_4)$ The exponent *p* in (2) is in the range
  $$\begin{aligned} 1<p<\frac{d+4}{4}. \end{aligned}$$
  This is to ensure that the holes are large enough that we are able to effectively estimate the intersections between the surface $\Gamma$ and the holes $T_\varepsilon$, of size $a_\varepsilon$. See the discussion following the estimate (15). In particular, if $p=2$ then $d>4$.

These are the assumptions required for using the framework from [4], though the $(A_4)$ is stricter here.

### Main results

The following theorems contain the main results of the present paper.

#### Theorem 1

Suppose $\Gamma$ is a $C^2$ convex surface. Let $I_\varepsilon \subset [0,1)$ be an interval, let $Q'\subset \mathbb {R}^{d-1}$ be a cube and let

$$\begin{aligned} A_\varepsilon = \#\left\{ k'\in \mathbb {Z}^{n-1}\cap \varepsilon ^{-1}Q':\frac{g(\varepsilon k')}{\varepsilon }\in I_\varepsilon \quad (\text {mod}1)\right\} . \end{aligned}$$

Then

$$\begin{aligned} \left| \frac{A_\varepsilon }{N_\varepsilon }-|I_\varepsilon |\right| = O(\varepsilon ^{\frac{1}{3}}), \end{aligned}$$

where $$N_\varepsilon = \#\{k'\in \mathbb {Z}^{d-1}\cap \varepsilon ^{-1}Q'\}$$.

Next we establish an important approximation result. We use the notation $T_\varepsilon ^k = \varepsilon k+a_\varepsilon T$ and $\Gamma _\varepsilon ^k=\Gamma \cap T_\varepsilon ^k$.

#### Theorem 2

Suppose $\Gamma$ is a $C^2$ convex surface and $P_x$ a support plane of $\Gamma$ at the point $x\in \Gamma$. Then

- $\mathbf 1^\circ$ the *p*-capacity of $P_x^k=P_x\cap T^k_\varepsilon$ approximates ${\text {cap}}_p(\Gamma ^k_\varepsilon )$ as follows
  3 $$\begin{aligned} {\text {cap}}_p(\Gamma _\varepsilon ^k) = {\text {cap}}_p(P_{x}^k\cap \{a_\varepsilon T+\varepsilon k\}) + o(a_\varepsilon ^{d-p}), \end{aligned}$$
  where $$x\in \Gamma _\varepsilon ^k$$.
- $\mathbf 2^\circ$ Furthermore, if $P_1$ and $P_2$ are two planes that intersect $\{a_\varepsilon T+\varepsilon k\}$ at a point *x*, with normals $\nu _1,\nu _2$ satisfying $|\nu _1-\nu _2|\le \delta$ for some small $\delta >0$, then
  4 $$\begin{aligned} |{\text {cap}}_p(P_1\cap \{a_\varepsilon T+\varepsilon k\})-{\text {cap}}_p(P_2\cap \{a_\varepsilon T+\varepsilon k\})|\le c_\delta a_\varepsilon ^{d-p}, \end{aligned}$$
  where $$\lim _{\delta \rightarrow 0}c_\delta =0$$.

As an application of Theorems 1, 2 we have

#### Theorem 3

Let $u_\varepsilon$ be the solution of (2). Then $u_\varepsilon \rightharpoonup u$ in $W_0^{1,p}(\Omega )$ as $\varepsilon \rightarrow 0$, where *u* is the solution to

5 $$\begin{aligned} \min \left\{ {\int _{{\Omega }} | \nabla v|^{p} dx + \int _{{\Gamma \cap \Omega }} | (\phi - v)_{ + } |^{p} {\text {cap}}_{{p,\nu (x)}} (T)dH^{{d - 1}} + \int _{{\Omega }} f vdx:v \in W_{0}^{{1,p}} (\Omega )} \right\} . \end{aligned}$$

In (5), $\nu (x)$ is the normal of $\Gamma$ at $x\in \Gamma$ and ${\text {cap}}_{p,\nu (x)}(T)$ is the mean *p*-capacity of *T* with respect to the hyperplane $P_{\nu (x)}=\{y\in \mathbb {R}^ d:\nu (x)\cdot y=0\}$, given by

6 $$\begin{aligned} {\text {cap}}_{p,\nu (x)}(T) = \int _{-\infty }^ {\infty }{\text {cap}}_p(T\cap \{P_{\nu (x)}+t\nu (x)\})dt, \end{aligned}$$

where ${\text {cap}}_p(E)$ denotes *p*-capacity of *E* with respect to $\mathbb {R}^ d$.

Theorem 3 was proved by the authors in [4] under the assumption that $\Gamma$ is a hyper plane, which was in turn a generalization of the paper [5]. In a larger context, Theorem 3 contributes to the theory of homogenization in non-periodic perforated domains, in that the support of the obstacle, $\Gamma _\varepsilon$, is not periodic. Another class of well-studied non-periodic perforated domains, not including that of the present paper, is the random stationary ergodic domains introduced in [1]. In the case of stationary ergodic domains the perforations are situated on lattice points, which is not the case for the set $\Gamma _\varepsilon$. The perforations, i.e. the components of $\Gamma _\varepsilon$, have desultory (though deterministic by definition) distribution. For the periodic setting [2] is a standard reference.

The proof of Theorem 3 has two fundamental ingredients. First the structure of the set $\Gamma _\varepsilon$ is analysed using tools from the theory of uniform distribution, Theorem 1. We prove essentially that the components of $\Gamma _\varepsilon$ are uniformly distributed over $\Gamma$ with a good bound on the discrepancy. This is achieved by studying the distribution of the sequence

7 $$\begin{aligned} \{\varepsilon ^{-1}g(\varepsilon k')\}_{k'}, \end{aligned}$$

for *g* defined by (1) and $\varepsilon k'\in Q'$. Second, we construct a family of well-behaved correctors based on the result of Theorem 2.

The major difficulty that arises when $\Gamma$ is a more general surface than a hyperplane is to estimate the discrepancy of the distribution of (the components of) $\Gamma _\varepsilon$ over $\Gamma$, which is achieved through studying the discrepancy of $\{\varepsilon ^{-1}g(\varepsilon k')\}_{k'}$. For a definition of discrepancy, see Sect. 2. In the framework of uniform convexity we can apply a theorem of Erdös and Koksma which gives good control of the discrepancy.

## Discrepancy and the Erdös–Koksma theorem

In this section we formulate a general result for the uniform distribution of a sequence and derive a decay estimate for the corresponding discrepancy.

### Definition 1

The discrepancy of the first *N* elements of a sequence $\{s_j\}_{j=1}^\infty$ is given by

$$\begin{aligned} D_N=\sup _{I\subset (0,1]}\left| \frac{A_N}{N}-|I|\right| , \end{aligned}$$

where *I* is an interval, |*I*| is the length of *I* and $A_N$ is the number of $1\le j\le N$ for which $s_j\in I\quad (\text {mod}1)$.

We first recall the Erdös–Turán inequality, see Theorem 2.5 in [7], for the discrepancy of the sequence $\{s_j\}_{j=1}^\infty$

8 $$\begin{aligned} D_N\le \frac{1}{n}+\frac{1}{N}\sum _{k=1}^n \frac{1}{k} \left| \sum _{j=1}^Ne^{2\pi if(j)k}\right| \end{aligned}$$

where *n* is a parameter to be chosen so that the right hand side has optimal decay as $N\rightarrow \infty$. Observe that $s_j$ is the *j*-th element of the sequence which in our case is $s_j=f(j)$ for a given function *f* and $N=\left[ \frac{1}{\varepsilon }\right]$.

We employ the following estimate of Erdös and Koksma ([7], Theorem 2.7) in order to estimate the second sum in (8): let $a, b\in \mathbb N$ such that $0<a<b$ then one has the estimate

9 $$\begin{aligned} \left| \sum _{j=1}^Ne^{2\pi if(j)k}\right| \le (|F_k'(b)-F_k'(a)|+2)\left( 3+\frac{1}{\sqrt{\rho }}\right) \end{aligned}$$

where $F_k(t) = kf(t)$ and $F_k''(t)\ge \rho >0$ for some positive number $\rho .$ In order to apply this result to our problem we first need to reduce the dimension of (7) to one. To do so let us assume that the obstacle $\Gamma$ is given as the graph of a function $x_d=g(x')$ where *g* is strictly convex $C^2$ function such that

10 $$\begin{aligned} c_0\delta _{\alpha , \beta }\le D_{x_\alpha x_\beta }g(x')\le C_0\delta _{\alpha , \beta }, \quad 1\le \alpha , \beta \le d-1 \end{aligned}$$

for some positive constants $c_0<C_0$.

Next we rescale the $\varepsilon$-cells and consider the normalised problem in the unit cube $[0, 1]^d$. The resulting function is $f(j)=\frac{g(\varepsilon j)}{\varepsilon }, j\in \mathbb {Z}^{d-1}$.

If $d=2$ then we can directly apply (9) to the scaled function *f* above. Otherwise for $d>2$ we need an estimate for the multidimensional discrepancy in terms of $D_N$ introduced in Definition 1, a similar idea was used in [4] for the linear obstacle. Suppose for a moment that this is indeed the case. Then we can take $F_k(t)=kf(t)$ in (9) and noting

11 $$\begin{aligned} D_{x_\alpha }f(x')=kD_\alpha g(\varepsilon x'), \quad D_{x_\alpha }^2f(x')=k\varepsilon D_\alpha ^2 g(\varepsilon x')\ge k\varepsilon c_0, \quad 1\le \alpha \le d-1 \end{aligned}$$

one can proceed as follows

$$\begin{aligned} \left| \sum _{j=1}^Ne^{2\pi if(j)k}\right|\le & {} (|kD_{x_\alpha }g(\varepsilon N)-kD_\alpha g(\varepsilon )|+2)\left( 3+\frac{1}{\sqrt{k\varepsilon c_0}}\right) \nonumber \\ {}\le & {} (k\varepsilon C_0(N-1)+2)\left( 3+\frac{1}{\sqrt{k\varepsilon c_0}}\right) \nonumber \\ {}\le & {} k\left( \varepsilon C_0(N-1)+\frac{2}{k}\right) \left( 3+\frac{1}{\sqrt{k\varepsilon c_0}}\right) \nonumber \\\le & {} k\left( \varepsilon C_0(N-1)+\frac{2}{k}\right) \left( 3+{\sqrt{\frac{N}{kc_0}}}\right) \nonumber \\\le & {} \lambda k\left( 1+\sqrt{\frac{N}{k}}\right) \end{aligned}$$

for some tame constant $\lambda >0$ independent of $\varepsilon , k$. Plugging this into (8) yields

$$\begin{aligned} D_N\le & {} \frac{1}{n}+\frac{\lambda }{N}\sum _{k=1}^n\left( 1+\sqrt{\frac{N}{k}}\right) \\\nonumber= & {} \frac{1}{n} +\frac{\lambda n}{N}+ \frac{\lambda }{\sqrt{N}} \sum _{k=1}^n \frac{1}{\sqrt{k}}\\\nonumber\le & {} \frac{1}{n}+\overline{\lambda }\sqrt{\frac{n}{N}}\left( 1+\sqrt{\frac{n}{N}}\right) \end{aligned}$$

for another tame constant $\overline{\lambda }>0$. Now to get the optimal decay rate we choose $\frac{1}{n} =\sqrt{\frac{n}{N}}$ which yields $N=n^3$ and hence

$$\begin{aligned}n=N^{\frac{1}{3}}\approx \frac{1}{\varepsilon ^{\frac{1}{3}}}\end{aligned}$$

and we arrive at the estimate

12 $$\begin{aligned} D_N = O( \varepsilon ^{\frac{1}{3}}). \end{aligned}$$

### Proof of Theorem 1

#### Proof

Suppose $$Q' $$ is a cube of size *r*. Then there is a cube $$Q''\subset \mathbb {R}^{d-2}$$ such that $$Q' = [\alpha ,\beta ]\times Q'$$, $$\beta -\alpha =r$$. We may rewrite $$A_\varepsilon $$ as

$$\begin{aligned} A_\varepsilon&=\sum _{k''\in \varepsilon ^{-1}Q''\cap \mathbb {Z}^{d-2}}\#\left\{ k_1\in \mathbb {Z}:a\le k_1\le b\text { and }\varepsilon ^{-1}g(\varepsilon k_1+\varepsilon k'')\in I_\varepsilon \quad (\text {mod}1)\right\} , \end{aligned}$$

where $(k_1,k'')=k'$, *a*, *b* are the integer parts of $\varepsilon ^{-1}\alpha$ and $\varepsilon ^{-1}\beta$ respectively and $|(b-a)- \varepsilon ^{-1}r|\le 1$. We also note that $N_\varepsilon = (\varepsilon ^{-1}r)^{d-1}+O(\varepsilon ^{-1}r)^{d-2}$. Consider

$$\begin{aligned} A_\varepsilon ^1(k'')=\#\left\{ k_1\in \mathbb {Z}:a\le k_1\le b\text { and }\varepsilon ^{-1}g(\varepsilon k_1+\varepsilon k'')\in I_\varepsilon \quad (\text {mod}1)\right\} . \end{aligned}$$

Then we have

13 $$\begin{aligned} \frac{A_\varepsilon }{N_\varepsilon }-|I_\varepsilon |&=\frac{1}{(\varepsilon ^{-1}r)^{d-2}}\sum _{k''\in \varepsilon ^{-1}Q''\cap \mathbb {Z}^{d-2}}\frac{A_\varepsilon ^1(k'')}{(\varepsilon ^{-1}r)}-|I_\varepsilon |. \end{aligned}$$

For each $k''$ the function $h:s\rightarrow \varepsilon ^{-1}g(\varepsilon s+\varepsilon k'')$ satisfies $|h'(s)|\le C_1$ and $h''(s)\ge \rho \varepsilon$ for $a\le s\le b$. Thus we may apply the Erdös-Koksma Theorem as described above and conclude that

$$\begin{aligned} \left| \frac{A_\varepsilon ^1(k'')}{(\varepsilon ^{-1}r)}-|I_\varepsilon |\right| \le C\varepsilon ^{\frac{1}{3}}. \end{aligned}$$

It follows that the modulus of the left hand side of (13) is bounded by $C\varepsilon ^{\frac{1}{3}}$, proving the theorem. $\square$

## Correctors

The purpose of this section is to construct a sequence of correctors that satisfy the hypotheses given below. Once we have established the existence of these correctors, the proof of the Theorem 3 is identical to the planar case treated in [4].

- $\mathbf H1$ $0\le w_\varepsilon \le 1$ in $\mathbb {R}^d$, $w_\varepsilon =1$ on $\Gamma _\varepsilon$ and $w_\varepsilon \rightharpoonup 0$ in $W^{1,p}_{{\text {loc}}}(\mathbb {R}^d)$,
- $$\mathbf H2$$ $$\int _\Omega |\nabla w_\varepsilon |^pfdx\rightarrow \int \limits _{\Gamma } f(x){\text {cap}}_{p,\nu _x}d\mathcal H^{d-1}$$, for any $$f\in W_0^{1,p}(\Omega )\cap L^\infty (\Omega )$$,
- $\mathbf H3$ (weak continuity) for any $\phi _\varepsilon \in W_0^{1,p}(\Omega )\cap L^\infty (\Omega )$ such that
  $$\begin{aligned} \left\{ \begin{array}{l} \sup \limits _{\varepsilon >0}\Vert \phi _\varepsilon \Vert _{L^\infty (\Omega )}<\infty ,\\ \phi _\varepsilon =0 \text { on } \Gamma _\varepsilon \text { and } \phi _\varepsilon \rightharpoonup \phi \in W_0^{1,p}(\Omega ), \end{array}\right. \end{aligned}$$
  we have
  $$\begin{aligned} \langle -\Delta _p w_\varepsilon , \phi _\varepsilon \rangle \rightarrow \langle \mu , \phi \rangle \end{aligned}$$
  with 14 where ${\text {cap}}_{p,\nu (x)}$ is given by (6) and  is the restriction of $s-$dimensional Hausdorff measure on $\Gamma$.

Setting $\Gamma _\varepsilon ^k:=\Gamma \cap \{a_\varepsilon T+\varepsilon k\}\ne \emptyset$, we define $w_\varepsilon ^k$ by

$$\begin{aligned} \Delta _p w_\varepsilon ^k&=0\quad \text { in }B_{\varepsilon / 2}(\varepsilon k){\setminus } \Gamma _\varepsilon ^k,\\ \nonumber w_\varepsilon ^k&=0\quad \text { on } \partial B_{\varepsilon / 2}(\varepsilon k),\\ \nonumber w_\varepsilon ^k&= 1\quad \text { on }\Gamma _\varepsilon ^k. \end{aligned}$$

Then it follows from the definition of ${\text {cap}}_p$ [3] that

$$\begin{aligned} \int _{B_{\varepsilon /2}(\varepsilon k)}|\nabla w_\varepsilon ^k|^pdx = {\text {cap}}_p(\Gamma _\varepsilon ^k) + o(a_\varepsilon ^{d-p}). \end{aligned}$$

Indeed, we have

$$\begin{aligned} {\text {cap}}_p(\Gamma _\varepsilon ^k, B_{\varepsilon /2}(\varepsilon k) )= & {} \inf \left\{ \int _{B_{\varepsilon /2}}|\nabla w|^p : w\in W^{1,p}_0(B_{\varepsilon /2}(\varepsilon k)) \ \mathrm{and} \ w=1 \ \mathrm{on } \ \Gamma _\varepsilon ^k \right\} \\\nonumber= & {} a_\varepsilon ^{d-p}\inf \left\{ \int _{B_{\varepsilon /{2a_\varepsilon }}}|\nabla w|^p : w\in W^{1,p}_0(B_{\varepsilon /2a_\varepsilon } \ \mathrm{and} \ w=1 \ \mathrm{on } \ \frac{1}{a_\varepsilon }\Gamma _\varepsilon ^k \right\} \\\nonumber= & {} a_\varepsilon ^{d-p}\left( {\text {cap}}_p\left( \frac{1}{a_\varepsilon }\Gamma _\varepsilon ^k\right) +o(1)\right) \\\nonumber= & {} {\text {cap}}_p(\Gamma _\varepsilon ^k)+o(a_\varepsilon ^{d-p}). \end{aligned}$$

Note that ${\text {cap}}_p(\Gamma _\varepsilon ^k)=O(a_\varepsilon ^{d-p})$ since $\Gamma _\varepsilon ^k=\Gamma \cap \{\varepsilon k+ a_\varepsilon T\}$ and ${\text {cap}}_p(t E)=t^{d-p}{\text {cap}}_p(E)$ if $t\in \mathbb {R}_+$ and $E\subset \mathbb {R}^d$. If $Q'$ is a cube in $\mathbb {R}^{d-1}$, the components of $\Gamma _\varepsilon \cap Q'\times \mathbb {R}$ are of the form $\Gamma _\varepsilon ^k=\Gamma \cap \{(\varepsilon k',\varepsilon k_d)+a_\varepsilon T\}$ for $\varepsilon k'\in Q'$. In particular, $\Gamma _\varepsilon ^ k\ne \emptyset$ if and only if $\varepsilon ^{-1} g(\varepsilon k')\in I_\varepsilon \quad (\text {mod}1)$ where $|I_\varepsilon |=O(a_\varepsilon /\varepsilon )$. Thus Theorem 1 tells us that the number of components of $\Gamma _\varepsilon \cap Q'\times \mathbb {R}$ equals $A_\varepsilon = |I_\varepsilon |N_\varepsilon +N_\varepsilon O(\varepsilon ^{\frac{1}{3}})$, or explicitly

15 $$\begin{aligned} \left| \frac{\frac{A_\varepsilon }{N_\varepsilon }}{\frac{a_\varepsilon }{\varepsilon }}-1\right| =\frac{{O}(\varepsilon ^{\frac{1}{3}})}{\frac{a_\varepsilon }{\varepsilon }}. \end{aligned}$$

Here we need to have $\varepsilon ^{1/3}=o(|I_\varepsilon |)$, which is equivalent to $(A_4)$. Since

$$\begin{aligned}\int _{B_{\varepsilon /2}(\varepsilon k)}|\nabla w_\varepsilon ^k|^pdx = {\text {cap}}_p(\Gamma _\varepsilon ^k) + o(a_\varepsilon ^{d-p}),\end{aligned}$$

we get

$$\begin{aligned} \int _{\mathbb {R}\times Q'}|\nabla w_\varepsilon |^ pdx\le C(|I_\varepsilon |N_\varepsilon {\text {cap}}_p(\Gamma _\varepsilon ^k))\le C\frac{a_\varepsilon }{\varepsilon }\varepsilon ^ {1-d}|Q'|a_\varepsilon ^ {n-p}=C|Q'|. \end{aligned}$$

Thus $\int _K|\nabla w_\varepsilon |^p$ is uniformly bounded on compact sets *K*. Since $w_\varepsilon (x)\rightarrow 0$ pointwise for $x\not \in \Gamma$, $\mathbf H1$ follows.

When verifying $\mathbf H_2$ and $\mathbf H_3$ we will only prove that

16 $$\begin{aligned} \lim _{\varepsilon \rightarrow 0}\int _Q|\nabla w_\varepsilon |^pdx= \int _{\Gamma \cap Q}c_{\nu (x)}d\mathcal {H}^{d-1}(x), \quad \text {for all cubes }Q\subset \mathbb {R}^d. \end{aligned}$$

Once this has been established the rest of the proof is identical to that given in [4].

## Proof of Theorem 2

### Proof

$\mathbf 1^\circ$ Set $R_\varepsilon =\frac{\varepsilon }{2a_\varepsilon }\rightarrow \infty$, then after scaling we have to prove that

17 $$\begin{aligned} \int _{B_{R_\varepsilon }}|\nabla v_1|^p-\int _{B_{R_\varepsilon }}|\nabla v_2|^p=o(1) \end{aligned}$$

uniformly in $\varepsilon$ where

$$\begin{aligned} \Delta _p v_i&=0\quad \text { in } B_{R_\varepsilon }{\setminus } S_i,\\ \nonumber v_i&=0\quad \text { on } \partial B_{R_\varepsilon },\\ \nonumber v_i&= 1\quad \text { on }S_i. \end{aligned}$$

and $$S_1=\frac{1}{a_\varepsilon }\Gamma ^k_\varepsilon , S_2=\frac{1}{a_\varepsilon }P_{x}$$.

We approximate $v_i$ in the domain $B_{R_\varepsilon }{\setminus } D^t_i$ with $D^t_i$ being a bounded domain with smooth boundary and $D^t_i\rightarrow S_i$ as $t\rightarrow 0$ in Hausdorff distance. Consider

$$\begin{aligned} \Delta _p v_i^t&=0\quad \text { in } B_{R_\varepsilon }{\setminus } D^t_i, \\ \nonumber v_i^t&=0\quad \text { on } \partial B_{R_\varepsilon },\\ \nonumber v_i^t&= 1\quad \text {on }\partial D^t_i. \end{aligned}$$

Observe that $\int _{B_{R_\varepsilon }{\setminus } D_i^t}|\nabla v_i^t|^{p}, i=1, 2$ remain bounded as $t\rightarrow 0$ thanks to Caccioppoli’s inequality. Indeed, $w=(1-v_i^t)\eta \in W^{1, p}_0(B_5{\setminus } D_i^t)$ where $\eta \in C_0^\infty (B_5)$ such that $0\le \eta \le 1$ and $\eta \equiv 1$ in $B_3$. Using *w* as a test function we conclude that

$$\begin{aligned}\int _{B_5{\setminus } D_i^t}|\nabla v_i^t|^p\eta =\int _{B_5{\setminus } D_i^t}|\nabla v_i^t|^{p-2}\nabla v_i^t\nabla \eta (1-v_i^t).\end{aligned}$$

Since $\eta \equiv 1$ in $B_3$ then applying Hölder inequality we infer that $\int _{B_3{\setminus } D_i^t}|\nabla v_i^t|^p\le C\int _{B_5}(1-v_i^t)^p$. In $B_{R_\varepsilon }{\setminus } B_2$ the $L^p$ we compare $W(x)=|x/2|^{\frac{p-d}{p-1}}$ with $v_i$. Note that our assumption $A_4$ implies that $p<d$. Moreover, since *W* is *p*-harmonic in $B_{R_\varepsilon }{\setminus } B_2$ then the comparison principle yields $v_i\le W$ in $B_{R_\varepsilon }{\setminus } B_2$. From the proof of Caccioppoli’s inequality above choosing non-negative $\eta \in C^\infty (\mathbb {R}^d)$ such that $\eta \equiv 0$ in $B_2$, $\frac{1}{2}\le \eta \le 1$ in $B_{R_\varepsilon }{\setminus } B_3$, and $\eta =1$ in $\mathbb {R}^d{\setminus } B_{R_\varepsilon }$ and using $\eta v_i\in W_0^{1, p}(B_{R_\varepsilon }{\setminus } B_2)$ as a test function we infer

$$\begin{aligned}\int _{B_{R_\varepsilon }{\setminus } B_3}|\nabla v_i|^p\le \frac{C}{R^p_\varepsilon }\int _{B_{R_\varepsilon }{\setminus } B_2}v_i^p\le \frac{C}{R_\varepsilon ^{\frac{1}{p-1}}}\rightarrow 0 \quad \text {as}\ \varepsilon \rightarrow 0\end{aligned}$$

where the last bound follows from the estimate $v_i\le W$. Combining these estimates we infer

18 $$\begin{aligned} \Vert v_i^t\Vert _{W^{1, p}(B_{R_\varepsilon })}\le K, \quad i=1,2 \end{aligned}$$

for some tame constant *K* independent of *t* and $\varepsilon$. Thus, by construction $v^t_i\rightharpoonup v_i$ weakly in $W^{1, p}_0(B_{R_\varepsilon })$.

Let $\psi \in C^\infty (\mathbb {R}^d)$ such that ${\text {supp}}\psi \supset D_1^t\cup D_2^t$ and $\psi \equiv 1$ in $\mathbb {R}^d{\setminus } B_2$. Then the function $\psi (v_1^t-v_2^t)\in W^{1,p}_0(B_{R_\varepsilon })$ and it vanishes on ${\text {supp}}\psi \supset D_1^t\cup D_2^t$. Thus we have

$$\begin{aligned}&\int _{B_{R_\varepsilon }}(\nabla v_1^t|\nabla v_1^t|^{p-2}-\nabla v_2^t|\nabla v_2^t|^{p-2})(\nabla v_1^t-\nabla v_2^t)\psi \\&\quad =-\int _{B_{R_\varepsilon }}(\nabla v_1^t|\nabla v_1^t|^{p-2}-\nabla v_2^t||\nabla v_2^t|^{p-2})(v_1^t- v_2^t)\nabla \psi \end{aligned}$$

Note that $v_1^t-v_2^t=0$ on $D^t_1\cap D_2^t$. Choosing a sequence $\psi _n$ such that $1-\psi _m$ converges to the characteristic function $\chi _{D_1^t\cup D_2^t}$ of the set $D_1^t\cup D_2^t$ we conclude

19 $$\begin{aligned} \int _{B_{R_\varepsilon }}(\nabla v_1^t|\nabla v_1^t|^{p-2}-\nabla v_2^t|\nabla v_2^t|^{p-2})(\nabla v_1^t-\nabla v_2^t)= J_1+J_2 \end{aligned}$$

where

$$\begin{aligned} J_1= & {} \int _{\partial D_1^t} (1- v_2^t)[\partial _\nu v_1^t|\nabla v_1^t|^{p-2}-\partial _\nu v_2^t|\nabla v_2^t|^{p-2}], \\\nonumber J_2= & {} \int _{\partial D_2^t}(v_1^t- 1)[\partial _\nu v_1^t|\nabla v_1^t|^{p-2}-\partial _\nu v_2^t|\nabla v_2^t|^{p-2}]. \end{aligned}$$

Notice that on $\partial D^t_i$ we have that $\nu =-\frac{\nabla \psi _m}{|\nabla \psi _m|}$ is the unit normal pointing inside $D^t_i$. We denote $n=-\nu$ and then we have that

$$\begin{aligned} -\int _{\partial D_1^t} (1- v_2^t)\partial _\nu v_2^t|\nabla v_2^t|^{p-2}= & {} \int _{\partial D_1^t} (1- v_2^t)\partial _n v_2^t|\nabla v_2^t|^{p-2}\\\nonumber= & {} \int _{\partial (D_1^t\cap D^t_2)} (1- v_2^t)\partial _n v_2^t|\nabla v_2^t|^{p-2}\\\nonumber= & {} \int _{D_1^t{\setminus } D_2^t} \text {div}((1-v_2^t)\nabla v_2^t|\nabla v_2^t|^{p-2})\\\nonumber= & {} -\int _{D_1^t{\setminus } D_2^t} |\nabla v_2^t|^p, \end{aligned}$$

and similarly

$$\begin{aligned} \int _{\partial D_2^t}(v_1^t- 1)\partial _\nu v_1^t|\nabla v_1^t|^{p-2}=-\int _{D_2^t{\setminus } D_1^t} |\nabla v_1^t|^p . \end{aligned}$$

Setting

20 $$\begin{aligned} I=\int _{B_{R_\varepsilon }}(\nabla v_1^t|\nabla v_1^t|^{p-2}-\nabla v_2^t|\nabla v_2^t|^{p-2})(\nabla v_1^t-\nabla v_2^t) \end{aligned}$$

and returning to (19) we infer

$$\begin{aligned} I= & {} -\int _{D_1^t{\setminus } D_2^t} |\nabla v_2^t|^p-\int _{D_2^t{\setminus } D_1^t} |\nabla v_1^t|^p+ \int _{\partial D_1^t} (1- v_2^t)\partial _\nu v_1^t|\nabla v_1^t|^{p-2} \\&\quad - \int _{\partial D_2^t}(v_1^t- 1)\partial _\nu v_2^t|\nabla v_2^t|^{p-2}\\\le & {} \int _{\partial D_1^t} (1- v_2^t)\partial _\nu v_1^t|\nabla v_1^t|^{p-2} - \int _{\partial D_2^t}(v_1^t- 1)\partial _\nu v_2^t|\nabla v_2^t|^{p-2}\\\le & {} \sup _ {D_1^t}(1-v_2^t)\int _{\partial D_1^t} |\partial _\nu v_1^t||\nabla v_1^t|^{p-2} +\sup _ {D_2^t}(1-v_1^t) \int _{\partial D_2^t}|\partial _\nu v_2^t||\nabla v_2^t|^{p-2}.\\ \end{aligned}$$

But on $\partial D_i^t$ we have $\partial _\nu v_i^t\ge 0$ ($\nu$ points inside $D_i^t$) because $v_i^t$ attains its maximum on $\partial D_i^t$. Thus we can omit the absolute values of the normal derivatives and obtain

$$\begin{aligned} I\le & {} \sup _ {D_1^t}(1-v_2^t)\int _{\partial D_1^t} \partial _\nu v_1^t|\nabla v_1^t|^{p-2} +\sup _ {D_2^t}(1-v_1^t) \int _{\partial D_2^t}\partial _\nu v_2^t|\nabla v_2^t|^{p-2}\\\nonumber= & {} \sup _ {D_1^t}(1-v_2^t)\int _{B_{R_\varepsilon }{\setminus } D_1^t} \text {div}(v_1\nabla v_1^t|\nabla v_1^t|^{p-2}) +\sup _ {D_2^t}(1-v_1^t) \int _{B_{R_\varepsilon }{\setminus } D_2^t}\text {div}(v_2\nabla v_2^t|\nabla v_2^t|^{p-2})\\\nonumber= & {} \sup _ {D_1^t}(1-v_2^t)\int _{B_{R_\varepsilon }{\setminus } D_1^t}|\nabla v_1^t|^{p} +\sup _ {D_2^t}(1-v_1^t) \int _{B_{R_\varepsilon }{\setminus } D_2^t}|\nabla v_2^t|^{p}. \end{aligned}$$

Recall that by Lemma 5.7 [6] there is a generic constant $M>0$ such that

21 $$\begin{aligned} (|\xi |^{p-2}\xi -|\eta |^{p-2}\xi )(\xi -\eta )\ge M\left\{ \begin{array}{lll} |\xi -\eta |^p &{}\quad \text {if}\ p>2,\\ |\xi -\eta |^2(|\xi |+|\eta |)^{p-2} &{}\quad \text {if}\ 1<p\le 2 \end{array} \right. \end{aligned}$$

for all $$\xi , \eta \in \mathbb {R}^d$$.

First suppose that $p>2$ then applying inequality (21) to (20) yields

$$\begin{aligned} I \ge M\int _{B_{R_\varepsilon }}|\nabla v_1^t-\nabla v_2^t|^{p}. \end{aligned}$$

As for the case $1<p\le 2$ then from (21) we have

$$\begin{aligned} I\ge M\int _{B_{R_\varepsilon }}|\nabla v_1^t-\nabla v_2^t|^2(|\nabla v_1^t|+|\nabla v_2^t|)^{p-2}. \end{aligned}$$

But, from Hölder’s inequality and (18) we get

22 $$\begin{aligned}&\int _{B_{R_\varepsilon }}|\nabla v_1^t-\nabla v_2^t|^p\nonumber \\&\quad = \int _{B_{R_\varepsilon }}|\nabla v_1^t-\nabla v_2^t|^p(|\nabla v_1^t|+|\nabla v_2^t|)^{\frac{p(p-2)}{2}}(|\nabla v_1^t|+|\nabla v_2^t|)^{-\frac{p(p-2)}{2}}\nonumber \\&\quad \le \left( \int _{B_{R_\varepsilon }}|\nabla v_1^t-\nabla v_2^t|^2(|\nabla v_1^t|+|\nabla v_2^t|)^{p-2}\right) ^{\frac{p}{2}} \left( \int _{B_{R_\varepsilon }}(|\nabla v_1^t|+|\nabla v_2^t|)^p\right) ^{1-\frac{p}{2}}\nonumber \\&\quad \le \left( \frac{I}{M}\right) ^{\frac{p}{2}} (2K)^{1-\frac{p}{2}}. \end{aligned}$$

Therefore, there is a tame constant $M_0$ such that for any $p>1$ we have

$$\begin{aligned}&\int _{B_{R_\varepsilon }}|\nabla v_1^t-\nabla v_2^t|^{p}\\&\quad \le M_0\left[ \sup _ {D_1^t}(1-v_2^t)\int _{B_{R_\varepsilon }{\setminus } D_1^t}|\nabla v_1^t|^{p} +\sup _ {D_2^t}(1-v_1^t) \int _{B_{R_\varepsilon }{\setminus } D_2^t}|\nabla v_2^t|^{p} \right] ^{\min (1, \frac{p}{2})}. \end{aligned}$$

Letting $t\rightarrow 0$ we get

23 $$\begin{aligned} \int _{B_{R_\varepsilon }}|\nabla v_1-\nabla v_2|^{p}\le & {} \liminf _{t\rightarrow 0} \int _{B_{R_\varepsilon }}|\nabla v_1^t-\nabla v_2^t|^{p}\nonumber \\\le & {} M_1\liminf _{t\rightarrow 0}\left[ \sup _ {D_1^t}(1-v_2^t) +\sup _ {D_2^t}(1-v_1^t) \right] ^{\min (1, \frac{p}{2})}. \end{aligned}$$

with some tame constant $M_1$.

Since $1-v_i^t$ are nonnegative *p*-subsolutions in $B_{R_\varepsilon }$, from the weak maximum principle, Theorem 3.9 [6] we obtain24Take a finite covering of $D^t_i$ with balls $B_r(z_k^i), z_k^i\in S_i, r=3a_\varepsilon , k=1, \dots , N$. Choose *t* small enough such that $D^t_j\subset \bigcup _{k=1}^N B_r(z_k^i)$ and applying (24) we obtain for $i,j\in \{1, 2\}$ with $i\not =j$
Since $\Vert v_i^t\Vert _{W^{1,p}(B_3)}\le C$ uniformly for all $t>0$ it follows that $v_i^t\rightarrow v_1$ strongly in $L^p(B_3)$ and $v_i$ is quasi-continuous. In other words, for any positive number $\theta$ there is a set $E_{\theta }$ such that ${\text {cap}}_p E_\theta <\theta$ and $v_i$ is continuous in $B_2{\setminus } E_\theta$. Notice that $E_\theta \subset S_1\cup S_2$ and hence $\mathcal H^d(E_\theta )=0$.

This yields25where $\omega _i(\cdot )$ is the modulus of continuity of $v_i$ on $B_{3}$ modulo the set $E_\theta$. Thus

$$\begin{aligned}\int _{B_{R_\varepsilon }}|\nabla v_1-\nabla v_2|^{p}\le C[\omega _{1}(6a_\varepsilon )+\omega _{2}(6a_\varepsilon )]^{p\min (1, \frac{p}{2})}.\end{aligned}$$

Hence (17) is established. Rescaling back and noting that $a_\varepsilon ^{d-p}\omega _i(a_\varepsilon )=o(a_\varepsilon ^{d-p})$ the result follows. Observe that $L^p$ norm of $\nabla v_i^t$ remains uniformly bounded in $B_{R_\varepsilon }$ by (18) and hence the moduli of quasi-continuity in, say, $B_3$ do not depend on the particular choice of $\Gamma _\varepsilon ^k$ or the tangent plane $P_{x}^k$.

$\mathbf{2^\circ }$ We recast the argument above but now for $S_1=\frac{1}{a_\varepsilon }P_1, S_2=\frac{1}{a_\varepsilon }P_{2}$. Squaring the inequality $|\nu _1-\nu _2|\le \delta$ we get that $2\sin \frac{\beta }{2}\le \delta$ where $\beta$ is the angle between $P_1$ and $P_2$. Since $\delta$ now measures the deviation of $v_1^t$ from 1 on $D_2^t$, (resp. $v_2^t$ on $D_1^t$) we conclude that the corresponding moduli of continuity of the limits $v_1, v_2$ (as $t\rightarrow 0$) modulo a set $E_\theta \subset S_1\cup S_2$ with small $p-$capacity depend on $\delta$, i.e.26where $B_r(z_k^i)$ provide a covering of $D_i^t$ as above but now, say, $r=6\delta$. Hence we can take $c_\delta =C(\omega _1(12\delta )+\omega _2(12\delta )).$ $\square$

## Proof of Theorem 3

We now formulate our result on the local approximation of total capacity (say in $Q'$) by tangent planes of $\Gamma$ and prove (16).

### Lemma 1

Fix a cube $Q'\subset \mathbb {R}^{d-1}$ such that if $x=(x',x_d)$ and $y=(y',y_d)$ belong to $\Gamma$ and $x',y'\in Q'$, then the normals $\nu _x,\nu _y$ of $\Gamma$ at *x* and *y* satisfy $|\nu _x-\nu _y|\le \delta$. Then for any $x=(x',x_d)\in \Gamma$ with $x'\in Q'$, there holds

$$\begin{aligned} \lim _{\varepsilon \rightarrow 0}\sum _{k\in \mathbb {Z}^n:k'\in \varepsilon ^{-1}Q'}\int _{B_\varepsilon ^k}|\nabla w_\varepsilon ^k|^pdx = [{\text {cap}}_{p,\nu _x}(T) + O(C_\delta )]\mathcal {H}^{d-1}(\Gamma _{Q'}), \end{aligned}$$

where $$\lim _{\delta \rightarrow 0}C_\delta =0$$ and $$\Gamma _{Q'}=\{x\in \Gamma :x'\in Q'\}$$.

### Proof

Fix $x\in \Gamma _{Q'}$ and let *P* be the plane $\{y:y\cdot \nu _x=0\}$, where $\nu _x$ is the normal of $\Gamma$ at *x*. Suppose $k=(k',k_d)\in \mathbb {Z}^d$, $\varepsilon k'\in Q'$ and let $P_{x^k}$ be the tangent plane to $\Gamma$ at $x^k=(\varepsilon k',g(\varepsilon k'))$. Then Theorem 2 $\mathbf 1^\circ$ tells us that

$$\begin{aligned} {\text {cap}}_p(\Gamma _\varepsilon ^k)={\text {cap}}_p(P_{x^k}\cap T_\varepsilon ^k)+o(a_\varepsilon ^{d-p}). \end{aligned}$$

If we set $P^k_\varepsilon = P+(-\varepsilon k',g(\varepsilon k'))$, then $P^k_\varepsilon$ will intersect the point $(\varepsilon k',g(\varepsilon k'))$. By assumption, $|\nu _x-\nu _{x^k}|\le \delta$, so

$$\begin{aligned} {\text {cap}}_p(P^k_\varepsilon \cap T_\varepsilon ^k)={\text {cap}}_p(P_{x^k}\cap T_\varepsilon ^k)+O(c_\delta a_\varepsilon ^{d-p}), \end{aligned}$$

by Theorem 2 $\mathbf 2^\circ$. This gives ${\text {cap}}_p(\Gamma _\varepsilon ^k)={\text {cap}}_p(P^k_\varepsilon \cap T_\varepsilon ^k)+O(c_\delta a_\varepsilon ^{d-p})$. Since, by Theorem 1, the sequence $\{\varepsilon ^{-1}g(\varepsilon k')\}_{k'\in \varepsilon ^{-1}Q'}$ is uniformly distributed mod 1 with discrepancy of order $\varepsilon ^{1/3}$, the rescaled planes $\varepsilon ^{-1}P^k_\varepsilon$ have the same distribution mod 1, i.e. they are translates of *P* and the translates have the same distribution. Using the proof of Lemma 4 of [4], we conclude that

$$\begin{aligned} \lim _{\varepsilon \rightarrow 0}\sum _{k\in \mathbb {Z}^n:k'\in \varepsilon ^{-1}Q'}{\text {cap}}_p(\{P_\varepsilon ^k \}\cap T_\varepsilon ^k) = {\text {cap}}_{p,\nu _x}(T)\mathcal {H}^{d-1}(P_{Q'}), \end{aligned}$$

where $P_{Q'}= \{x\in P:x'\in Q'\}$. Since we know that $\int _{B_\varepsilon ^k}|\nabla w_\varepsilon ^k|^pdx= {\text {cap}}_p(\Gamma _\varepsilon ^k)+o(a_\varepsilon ^{d-p})$, the result follows from the fact that $\mathcal {H}^{d-1}(\Gamma _{Q'})=(1+O(c_\delta ))\mathcal {H}^{d-1}(P_{Q'})$. $\square$

### Lemma 2

$$\begin{aligned} \lim _{\varepsilon \rightarrow 0}\int _Q|\nabla w_\varepsilon |^pdx = \int _{\Gamma \cap Q} {\text {cap}}_{p,\nu _x}(T)d\mathcal {H}^{d-1}. \end{aligned}$$

### Proof

The claim follows by decomposing the set $\{x'\in \mathbb {R}^{d-1}:(x',g(x'))\in \Gamma \cap Q\}$ into disjoint cubes $\{Q_j'\}$ that satisfy the hypothesis of Lemma 1. Since $\Gamma$ is $C^2$, we can find a finite number of disjoint cubes $\{Q_j\}_{j=1}^{N(\delta )}$, such that $\mathcal {H}^{d-1}(\Gamma \cap Q{\setminus } \cup _j Q_j\cap \Gamma )=0$ and $Q_j'$ is as in Lemma 1. For all $x\in \Gamma \cap Q_j$ we have $x=(x',g(x))$ for $x'\in Q_j'$, after interchanging coordinate axes if necessary. Thus

$$\begin{aligned} \lim _{\varepsilon \rightarrow 0}\int _Q|\nabla w_\varepsilon |^pdx&= \sum _j\lim _{\varepsilon \rightarrow 0}\sum _{k\in \mathbb {Z}^n:k'\in \varepsilon ^{-1}Q'_j}\int _{B_\varepsilon ^k}|\nabla w_\varepsilon ^k|^pdx\\&=\sum _{x^j\in Q_j'}[{\text {cap}}_{p,\nu _{x^j}}(T)+ O(C_\delta )]\mathcal {H}^{d-1}(\Gamma _{Q_j'}) \\&=\int _{\Gamma \cap Q}{\text {cap}}_{p,\nu (x)}(T)d\mathcal {H}^{d-1}+O(C_\delta ), \end{aligned}$$

where in the last step we used that ${\text {cap}}_{p,\nu (x)}(T)={\text {cap}}_{p,\nu _{x^j}}(T)+O(C_\delta )$ for all $x\in \Gamma _{Q_j'}$, by Lemma 1. Sending $\delta \rightarrow 0$ proves the lemma. $\square$

Having established Lemma 2, the rest of the proof of $\mathbf {H_2}$ and $\mathbf {H_3}$ is carried out precisely as in [4], with Lemma 2 above replacing Lemma 4 in [4]. The proof of Theorem 3 from **H** $_\mathbf 1$–**H** $_\mathbf 3$ is given in section 4 of [4] when $\Gamma$ is a hyper plane, and remains the same for the present case when $\Gamma$ is a convex surface.
